# Podoplanin, a Potential Therapeutic Target for Nasopharyngeal Carcinoma

**DOI:** 10.1155/2019/7457013

**Published:** 2019-06-20

**Authors:** Yen-Bin Hsu, Chi-Ying F. Huang, Kuan-Ting Lin, Yu-Lun Kuo, Ming-Chin Lan, Ming-Ying Lan

**Affiliations:** ^1^Department of Otolaryngology-Head and Neck Surgery, Taipei Veterans General Hospital, Taipei 11217, Taiwan; ^2^Institute of Clinical Medicine, National Yang-Ming University, Taipei 11221, Taiwan; ^3^Institute of Biopharmaceutical Sciences, National Yang-Ming University, Taipei 11221, Taiwan; ^4^Cold Spring Harbor Laboratory, Cold Spring Harbor, NY 11724, USA; ^5^Biotools, Co., Ltd, New Taipei City 22175, Taiwan; ^6^Department of Otolaryngology-Head and Neck Surgery, Taipei Tzu Chi Hospital, Buddhist Tzu Chi Medical Foundation, New Taipei City 23142, Taiwan; ^7^School of Medicine, Tzu Chi University, Hualien 97004, Taiwan; ^8^School of Medicine, National Yang-Ming University, Taipei 11221, Taiwan

## Abstract

**Introduction:**

The role of podoplanin (PDPN) in nasopharyngeal carcinoma (NPC) is still unknown. The aims of this study were to investigate the expression and role of PDPN in NPC cells.

**Materials and Methods:**

Immunofluorescence staining and functional tests were used to determine the effects of PDPN knockdown by siRNA in TW01 NPC cells. Microarray analysis was conducted to identify genes regulated by PDPN. The molecular mechanism of PDPN on NPC cells was further determined by Ingenuity Pathways Analysis (IPA).

**Results:**

PDPN was expressed in most TW01 NPC cells. PDPN knockdown by siRNA decreased NPC cell proliferation, migration, and invasion. The microarray data showed 63 upregulated genes and 12 downregulated genes following PDPN knockdown. The top 5 most upregulated genes analyzed by IPA were IFI27, IFI44L, IFI6, OAS1, and TRIM22, and the most relevant pathway was the interferon signaling pathway.

**Conclusions:**

To the best of our knowledge, this is the first report to show that knocking down PDPN leads to suppression of NPC cell proliferation, migration, and invasion. Our results suggest that PDPN may serve as a potential chemotherapeutic target for NPC treatment in the future.

## 1. Introduction

Nasopharyngeal carcinoma (NPC) is a common malignancy in South Asia but is rare in Western countries. Its annual incidence in southern China is > 20 cases per 100,000 people. The pathogenesis of NPC is related to genes, the Epstein-Barr virus (EBV), environment, and diet [1–5]. Currently, radiotherapy is the main method for treating NPC, and chemotherapy is added in cases with advanced stages. The five-year survival for early-stage NPC is quite high and nearly 90%. However, the prognosis of late-stage NPC is still unsatisfactory, with the 5-year overall survival rates ranging from 60% to 73% in the literature [6–8]. Similar to many other cancer types, local recurrence and distant metastases remain the main causes of treatment failure in NPC patients [1–5]. Thus, identifying new molecular markers may provide a way to improve the survival of these patients by developing a novel targeted therapy.

Podoplanin (PDPN), a 36- to 43-kDa transmembrane sialomucin-like glycoprotein, has three structural domains: a highly O-glycosylated (*α*2,3-sialic acid linked to galactose) extracellular domain, a hydrophobic transmembrane domain, and a short nine-amino-acid cytoplasmic tail [9]. It was given different names in several previous studies such as PA2.26 antigen, E11 antigen, gp38, T1a, and Aggrus and was finally called “podoplanin” because of its expression on kidney podocytes with association in the flattening of the podocyte foot [10]. The expression of PDPN has been characterized in many normal tissues including kidney podocytes, type-1 lung alveolar cells, basal keratinocytes, synovial fibroblasts, and lymphatic endothelial cells, and it is now widely used as a marker for lymphatic endothelial cells and fibroblastic reticular cells in lymphoid organs [11].

PDPN was suggested to play an important role in the development of several organs in mouse studies including the formation of the lymphatic vasculature, lung morphogenesis, and cardiac development. Several functions of PDPN have been found [12]. Its induction of platelet aggregation via interaction with the C-type lectin-like receptor 2 (CLEC-2) in platelets was found to be related to the differentiation of the lymphatic vasculature from the blood vascular system, the maintenance of high endothelial venule integrity in lymph nodes, and tumor-induced platelet aggregation [12]. Recently, PDPN was also found to be upregulated in a variety of tumors such as vascular tumors, malignant mesothelioma, tumors of the central nervous system (CNS), germ cell tumors, and squamous cell carcinomas [13, 14]. Moreover, several studies have revealed that PDPN is involved in the motility and metastasis of tumor cells [13, 15–20]. However, its role in NPC remains unclear.

To characterize the role of PDPN in NPC, we investigated the expression of PDPN in NPC cell lines and several functional studies were conducted in our study.

## 2. Materials and Methods

### 2.1. Cell Culture

The TW01 NPC cell line was kindly provided by Dr. Lin CT (Department of Pathology and Graduate Institute of Pathology, College of Medicine, National Taiwan University, Taiwan) [21, 22]. The cell line was derived from primary nasopharyngeal tumors from Chinese patients with* de novo *NPC. The NPC cell line was maintained in DMEM with 10% FBS at 37°C under 5% CO_2_.

### 2.2. siRNA Transfection

To knock down PDPN expression in NPC cells, the cells were first seeded on chamber slides or 96-well plates for 24 hours and then transfected with various concentrations of PDPN siRNA (Dharmacon, L-048117-01-0005, CO, USA) with Lipofectamine 3000 (Life Technologies, CA, USA) according to the instructions of the manufacturer. PDPN expression was analyzed by immunofluorescence 48 hours after siRNA transfer.

### 2.3. Immunofluorescence Staining of Cultured Cells

After the cells plated on chamber slides were confluent, the adherent cells were fixed for 20 minutes in 4% paraformaldehyde (Electron Microscopy Sciences Corp., Hatfield, PA, USA) at room temperature. Cells were then washed in PBS for 5 minutes three times, blocked in blocking buffer for 1 hour, and then incubated with mouse anti-human D2-40 monoclonal antibody (podoplanin; BioLegend, San Diego, CA, USA) overnight at 4°C. The D2-40 antibody was used as the primary antibody at a dilution of 1:40. For visualization, the secondary antibody, preabsorbed goat anti-mouse DyLight-488 (Abcam plc, Cambridge, UK), was used at a dilution of 1:500 at room temperature for 2 hours. Hoechst stain (Molecular Probes, Thermo Fisher Scientific Inc., Waltham, MA, USA) was used at a dilution of 1:10000 for 15 minutes to stain nuclei. Cells were then mounted using antifade fluorescent mounting media (DAKO, Carpinteria, CA, USA). Images were then acquired under a fluorescence microscope (Olympus Fluoview FV10i, USA) with Fluoview software (Olympus, USA).

### 2.4. WST-1 Cell Viability Test

The viability of the exposed cells was determined using the WST-1 cell proliferation reagent kit (Roche, Penzberg, Germany) according to the manufacturer's instructions. 1 × 10^4^ cells/well were seeded in a 96-well microplate for 24 hours. Cells were washed with PBS twice and exposed to the control or to various concentrations of PDPN siRNA in a humidified atmosphere (37°C and 5% CO_2_) for 3 days. The cells were incubated with 10 *μ*l WST-1 cell proliferation reagent for 2 hours, followed by using a microplate reader (Spectral Max 250) at 450 nm to measure the optical density.

### 2.5. Wound Healing Assay

Cells were plated in 48-well plates. When the cells grew to full confluency, a wound was created on the monolayer cells by scraping using a micropipette tip after cells had been treated with control or PDPN siRNA for 24 hours. The speed of wound closure was compared between the PDPN siRNA-treated groups and the control siRNA group. After wound incision and 24 hours later, photographs were taken under 100× magnification using phase contrast microscopy.

### 2.6. Cell Invasion Assay

Cell invasion assay was using a Transwell cell culture chamber (Millipore, Bedford, MA, USA) as described previously [23]. Briefly, a 8-*μ*m pore size polycarbonate filter was first coated with Matrigel, dried, and then reconstituted at 37°C with culture medium. 2 × 10^4^ cells in DMEM containing 10% FBS per chamber were added to the upper chamber, whereas culture medium containing 20% FBS was placed in the lower chamber. Then, 2 × 10^4^ cells in DMEM containing 10% FBS per chamber were added to the upper chamber. After cells were incubated with control or PDPN siRNA at 37°C for 48 hours, cells that had invaded the lower side of the filter were fixed in methanol, stained with DAPI, and five fields per chamber were then counted under a fluorescence microscope.

### 2.7. Microarray Hybridization and Analysis

RNA from the NPC cells treated with 200 nM PDPN siRNA was amplified and labeled with biotin using the Ovation Biotin RNA Amplification and Labeling System (NuGEN, San Carlos, CA, USA) according to the manufacturer's procedure. The fragmented, biotinylated cDNA was used for hybridization at 45°C for 17 hours with Affymetrix HG-U133 Plus 2.0 GeneChip microarrays (Affymetrix, Santa Clara, CA) as described by the manufacturer's recommendations. We scanned and digitized the hybridization signals using an Affymetrix 7G Gene Chip Scanner and GCOS Version 1.4.0.036 software, respectively. The raw expression data for differential expression analysis were normalized using the GCRMA package and the expression value for each probe was defined as the base 2 logarithm of the intensity in the samples. The microarray data were deposited in the GEO database (GEO accession number: GSE128502). We then used Ingenuity Pathway Analysis (IPA) to assign biological functions to genes and network analysis using the Ingenuity Pathways Knowledge Base (Ingenuity Systems, Inc., Redwood City, CA, USA).

### 2.8. Statistical Analysis

All experiments were carried out in triplicate, and at least three independent experiments were performed. The results are presented as the means ± SDs. Statistical comparisons of multigroup data were analyzed by ANOVA, followed by Sheffe's post-test using SPSS 12.0 software (SPSS Inc. Chicago, IL). A value of p<0.05 indicated statistical significance.

## 3. Results

**Figure 1:**
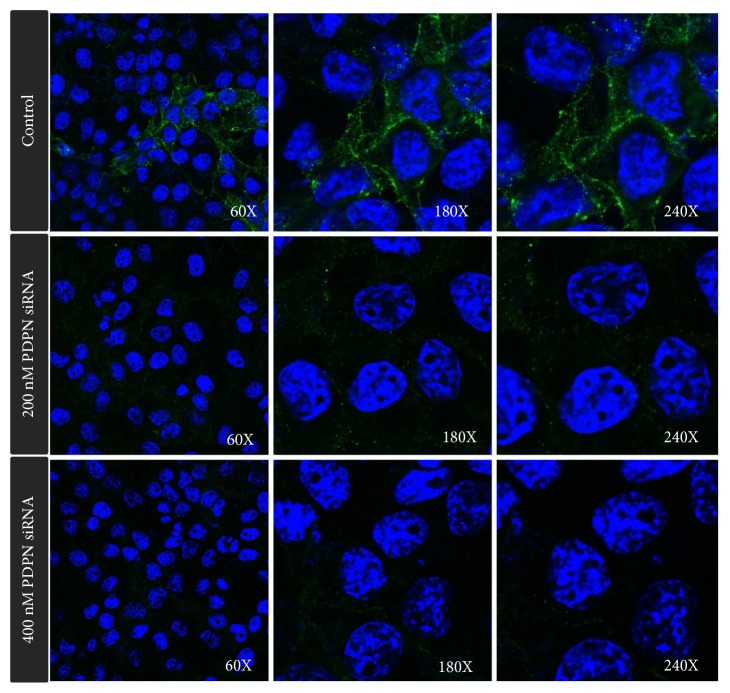
Expression of PDPN in TW01 NPC cells. PDPN expression was found in most TW01 NPC cells (upper row). The expression of PDPN decreased after cells were treated with PDPN siRNA (lower row). PDPN in green; Hoechst nuclear stain in blue.

**Figure 2:**
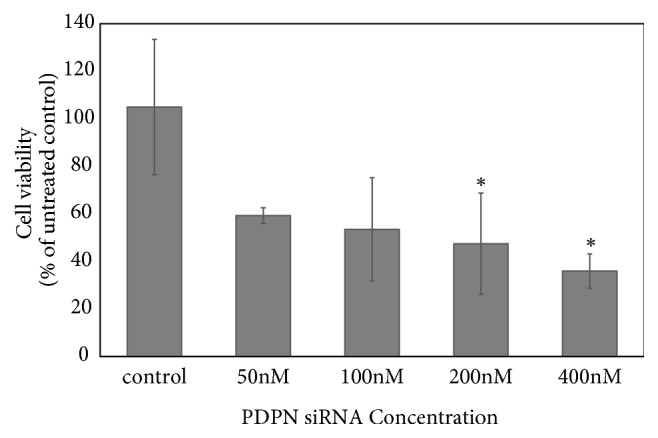
Function of PDPN in TW01 NPC cell proliferation. The WST-1 assay revealed that TW01 NPC cell proliferation was suppressed after cells were treated with PDPN siRNA. PDPN siRNA reduced cell viability in a concentration-dependent manner. Data are means ± SEs from three independent experiments. *∗*p < 0. 05 compared with the vehicle control group by ANOVA.

**Figure 3:**
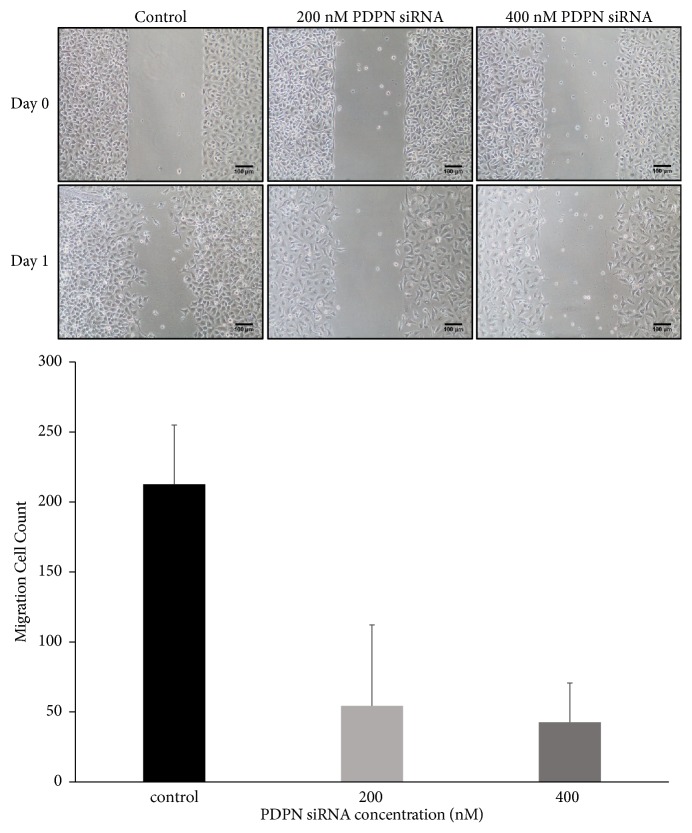
Function of PDPN in TW01 NPC cell migration. More cells migrated to the denuded area of the wounds in the control group (left) than in the cells transfected with 200 nM PDPN siRNA (middle) and 200 nM PDPN siRNA (right) 24 hours after the creation of the wound.

**Figure 4:**
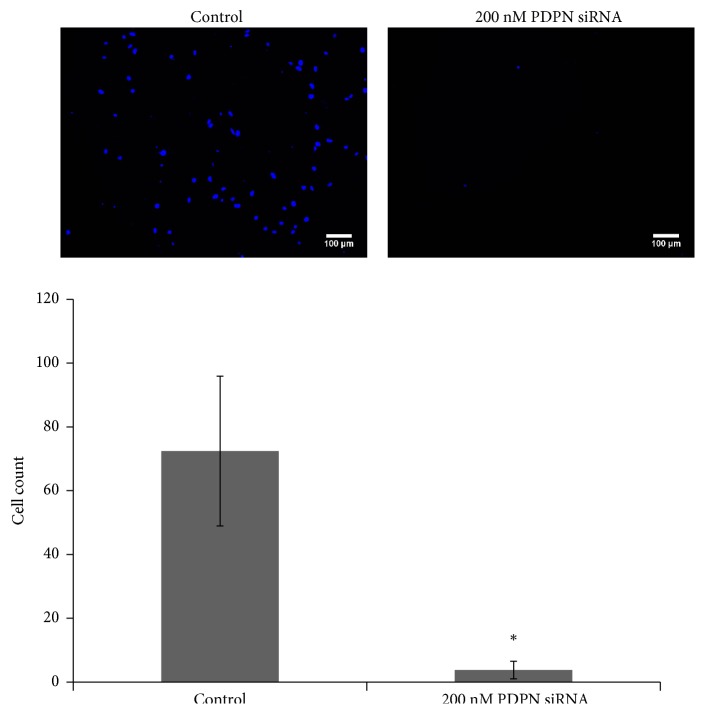
Function of PDPN in TW01 NPC cell invasion. Matrigel invasion assays of TW01 NPC cells in the control group and the PDPN siRNA group. Migrating cell numbers were counted on the opposite surfaces of filter membranes 48 hours after seeding. *∗*p < 0.001 compared with the vehicle control group by ANOVA.

Positive PDPN staining was observed in most TW01 NPC cells. The expression of PDPN decreased after cells were treated with PDPN siRNA (Figure 1). The WST-1 assay revealed that NPC TW01 cell proliferation was suppressed after cells were treated with PDPN siRNA (Figure 2). PDPN siRNA reduced cell viability in a concentration-dependent manner. The wound healing assay was used to observe whether PDPN played a role in cell motility. Indeed, we found that TW01 NPC cells transfected with 200 nM PDPN siRNA showed decreased cell migration compared to that of the control group (Figure 3). In addition, the Matrigel invasion assays revealed that the invasion of TW01 NPC cells decreased after the cells were transfected with 200 nM PDPN siRNA for 48 hours (Figure 4).

**Figure 5:**
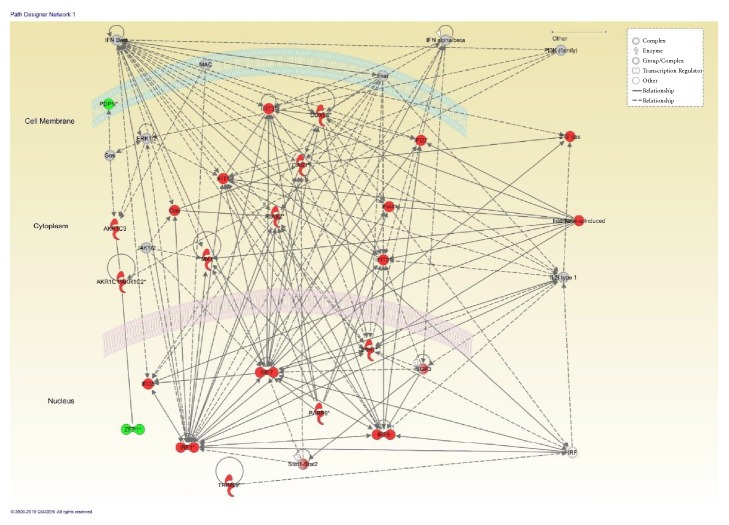
The most highly rated network analyzed with IPA. The genes shaded red are upregulated, and those shaded green are downregulated. All shaded genes are statistically significant. A dotted line means an indirect interaction between the two gene products, and a solid line represents a direct interaction.

To identify differentially expressed genes, the gene expression profiles of the NPC cell lines treated with and without 200 nM PDPN siRNA were compared. A total of 75 genes were differentially expressed by at least 2-fold, including 63 upregulated and 12 downregulated genes. The data were then analyzed using the functional analysis tool IPA. The Ingenuity Pathways Knowledge Base, which includes more than 49,000 datasets containing thousands of human, mouse, and rat genes, is used to assign biological functions to genes with IPA. The top 10 upregulated molecules, top 10 downregulated molecules, and top 5 transcription factors are listed in Tables 1, 2, and 3, respectively.  Table 4 lists the top 5 ingenuity canonical pathways involved in the effect of PDPN siRNA on NPC cells, including interferon signaling, the activation of IRF by cytosolic pattern recognition receptors, the role of pattern recognition receptors in the recognition of bacteria and viruses, role of PKR in interferon induction and antiviral response, and the role of RIG1-like receptors in antiviral innate immunity. The most highly rated network analyzed by IPA is shown in Figure 5.

## 4. Discussion

PDPN is a unique mucin-type transmembrane sialoglycoprotein that has been widely used as a lymphatic endothelial marker. Recently, it was found to be overexpressed in various cancers including lymphangioma, Kaposi sarcoma, hemangioendothelioma, epithelioid mesothelioma, seminoma, hemangioblastoma, glioblastoma multiforme, oral squamous cell carcinoma (OSCC), head and neck SCC, and squamous nonsmall cell lung cancer [13, 14, 24, 25]. The present study is the first to characterize PDPN expression in an NPC cell line.

PDPN is expressed by aggressive tumors with high invasive and metastatic potential [26]. Wicki et al. showed that PDPN-expressing cells were found at the invasion front in more than 80% human squamous cell carcinomas [15]. PDPN expression also predicted the prognosis of patients with oral SCC treated with neoadjuvant chemoradiotherapy [27]. In addition to its role in tumor cells, PDPN also plays a role in cancer-associated fibroblasts. PDPN-positive fibroblast infiltration significantly decreased overall survival, disease-free survival, and progression-free survival in lung cancer patients [28]. Katsumata et al. found that PDPN-positive cancer-associated fibroblasts enriched at the outer edge of breast tumors suppressed the proliferation of T cells in a nitric oxide-dependent manner [29].

PDPN promotes cell migration by interacting with the ezrin/radixin/moesin (ERM) protein family, which anchors the actin cytoskeleton [15]. Its involvement in actin remodeling of the cytoskeleton of tumor cells promotes tumor cell invasion by increasing cell motility and the formation of filopodia-like membrane protrusions [14]. The interaction between PDPN and CLEC-2 is postulated to regulate tumor invasion and metastasis [16–20]. PDPN knockdown could cause impaired cell spreading with reduced filopodia. In contrast, its overexpression could induce an increase in cellular protrusions and stress fibers with extensive parallel bundles [14]. Takeuchi et al. found that the overexpression of PDPN in malignant pleural mesothelioma cells expressing low levels of PDPN enhanced cell motility, while knocking down PDPN in malignant pleural mesothelioma cells expressing high levels of PDPN decreased cell motility. They proposed that PDPN-stimulated motility was mediated by the activation of the RhoA/ROCK pathway [30]. Another study suggested that PDPN mediates cytoskeletal remodeling and invasion via hierarchical crosstalk between PDPN, Cdc42, and MT1-MMP in the invadopodia [14]. EGF-Src-Cas pathway is another postulated mechanism through which PDPN is involved in cell migration, which results in the progression of oral SCC [31].

Ohta et al. revealed that TGF-*β* positively and negatively regulated the expression of PDPN [32]. Mei et al. found that ErbB3 binding protein-1 (Ebp1) served as a transcriptional activator to drive PDPN expression and contributed to oral tumorigenesis [33]. Tsuneki1 et al. found that PDPN-positive odontogenic tumor cells were located within areas of PCNA-positive cells and that integrin *β*1 was localized in the cell membrane of PDPN-positive cells in the intercellular space, whereas fibronectin and MMP-9 were deposited, indicating its close association with extracellular matrix signaling [34]. They later demonstrated that PDPN collaborated with CD44 in cell adhesion by tethering oral SCC cells to the hyaluronan-rich ECM to secondarily promote oral SCC cell proliferation [35]. Cioca et al. showed that podoplanin had multiple functions in HCC: tumorigenesis, lymphatic neovascularization, and tumor invasion [36]. Miyashita el al. revealed that the high clonal expansion capacity of podoplanin-positive tumor-initiating cell populations was the result of reduced cell death by podoplanin-mediated signaling. They proposed that podoplanin activity may be a therapeutic target for the treatment of squamous cell carcinomas [37].

In our study, the knockdown of PDPN repressed the proliferation, migration, and invasion of NPC cells. We further used microarray analysis followed by IPA analysis to identify PDPN-regulated genes. Our IPA data revealed the top 10 upregulated and top 10 downregulated molecules. Besides PDPN, many of these molecules, such as IFI6, IFI27, IFI44L, and BOP1, also have been reported to be associated with carcinogenesis and treatment in other types of cancers. IFI6 (interferon alpha inducible protein 6), which plays a critical role in the regulation of apoptosis, is induced by interferon. Another interferon alpha inducible protein, IFI27, has been found to be upregulated in some cancers. Moreover, IFI27 overexpression could induce epithelial-mesenchymal transition and promote migration, invasion, tumorigenicity, stemness, and drug resistance in ovarian cancer cells [38]. IFI44L (interferon induced protein 44 like) can affect cancer stemness, metastasis, and drug resistance in hepatocellular carcinoma (HCC). It is a novel tumor suppressor and an important prognostic marker of HCC [39]. BOP1 (block of proliferation 1) has been reported to play an oncogenic role in HCC by promoting epithelial to mesenchymal transition [40].

PDPN might be a potential target for antimetastatic therapy. Ochoa-Alvarez et al. demonstrated that targeting PDPN with a monoclonal antibody (NZ-1) and lectin (MASL) inhibited the migration of PDPN-expressing OSCC cells at nanomolar concentrations and inhibited cell viability at micromolar concentrations through caspase-independent nonapoptotic necrosis [41]. It has been proposed that tumor-related thrombosis, subsequent inflammation, and inflammation-induced cachexia are related to the CLEC-2-podoplanin interaction and that anti-podoplanin has the potential to prevent tumor metastasis and progression in cancer patients [42, 43]. In our study, we demonstrated that the transfection of NPC cells with PDPN siRNA truly decreased NPC cell proliferation and cell motility. It seems that PDPN will be a promising target for antitumor therapy in NPC in the future.

## 5. Conclusions

Our findings demonstrated PDPN expression in NPC and its involvement in NPC cell proliferation, migration, and invasion. PDPN has potential as a chemotherapeutic target for NPC treatment in the future.

## Figures and Tables

**Table 1:** Top 10 upregulated molecules analyzed by IPA.

Rank	Gene	Expression value
1	IFI27	7.041
2	IFI44L	5.551
3	IFI6	5.029
4	OAS1	4.580
5	TRIM22	3.315
6	IFITM1	3.130
7	OAS2	3.069
8	STAT1	2.809
9	CMPK2	2.793
10	OAS3	2.771

**Table 2:** Top 10 downregulated molecules analyzed by IPA.

Rank	Gene	Expression value
1	PDPN	-2.199
2	NALCN	-1.249
3	TMTC4	-1.220
4	ZFP1	-1.138
5	LINC00973	-1.114
6	JPH3	-1.101
7	BOP1	-1.060
8	MMP12	-1.043
9	RGL3	-1.042
10	SMIM2-AS1	-1.024

**Table 3:** Top 5 upstream regulators analyzed by IPA.

Rank	Gene	p-value	Predicted Activation
1	IFNL1	1.15E-61	Activated
2	IFNA2	1.24E-58	Activated
3	MAPK1	1.59E-42	Inhibited
4	Interferon alpha	1.78E-41	Activated
5	IRF7	2.50E-41	Activated

**Table 4:** Top 5 canonical pathways identified by IPA.

Rank	Canonical Pathways	p-value	Overlap
1	Interferon Signaling	5.53E-16	27.8% (10/36)
2	Activation of IRF by Cytosolic Pattern Recognition Receptors	3.44E-07	10.0% (6/60)
3	Role of Pattern Recognition Receptors in Recognition of Bacteria and Viruses	3.55E-07	5.5% (8/145)
4	Role of PKR in Interferon Induction and Antiviral Response	8.64E-04	7.5% (3/40)
5	Role of RIG1-like Receptors in Antiviral Innate Immunity	9.29E-04	7.3% (3/41)

## Data Availability

The data used to support the findings of this study are available from the corresponding author upon request.
